# Supplementary material for: Chloroplast Genomes and Phylogenetic Analysis of Three Carthamus (Asteraceae) Species
Source: Int J Mol Sci. 2023 Oct 26;24(21):15634. doi: 10.3390/ijms242115634 (PMC10648744; doi:10.3390/ijms242115634)
Supplement: Supplementary file 1 [file ijms-24-15634-s001.zip › Supplementary Figure.pdf]

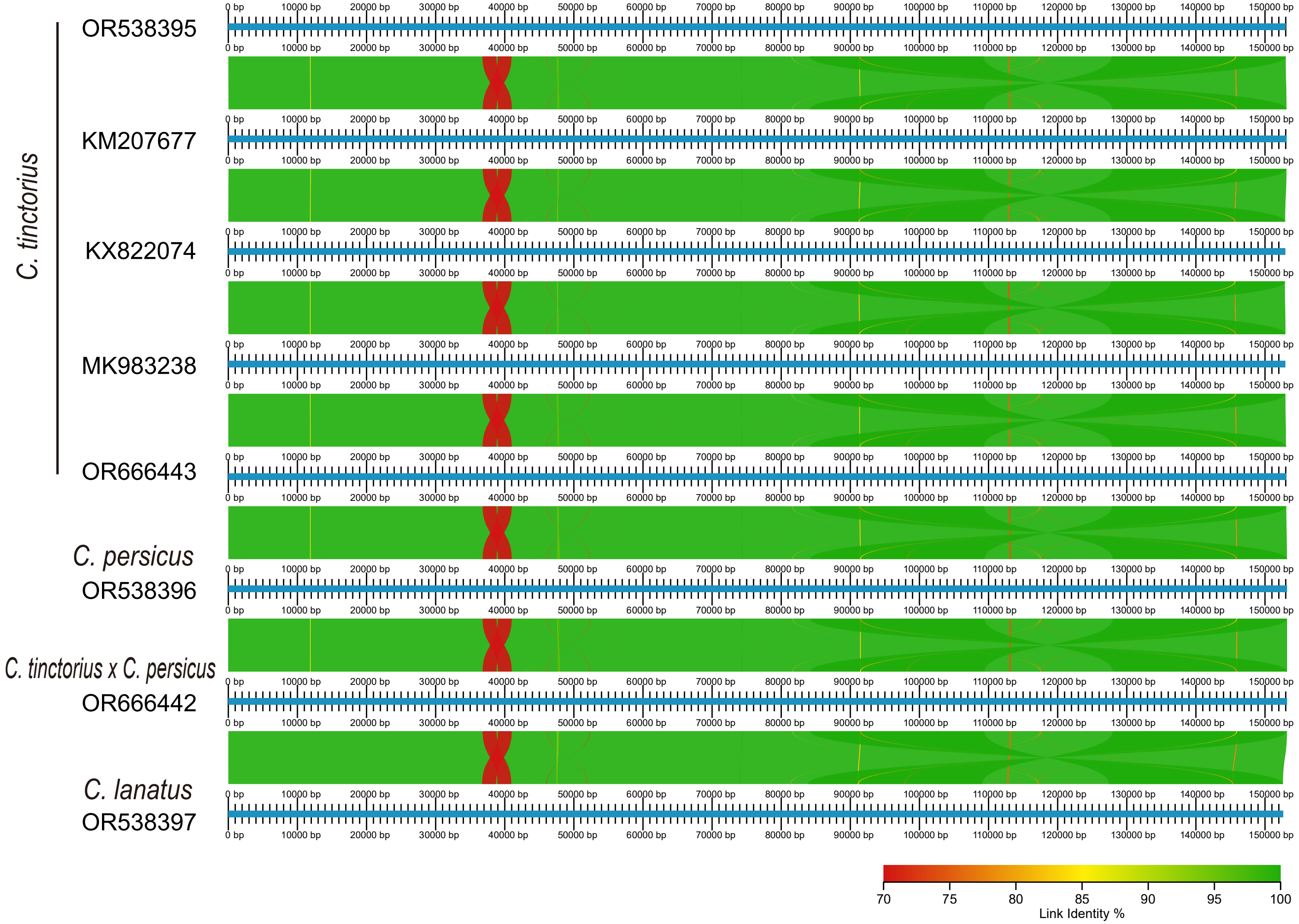

Figure S1. Genomic collinearity analysis of four *Carthamus* species. The gray-blue color indicates the entire chloroplast genome region, and the line connecting them indicates the chaining relationship. Chaining relationships are indicated in red to green from low to high.

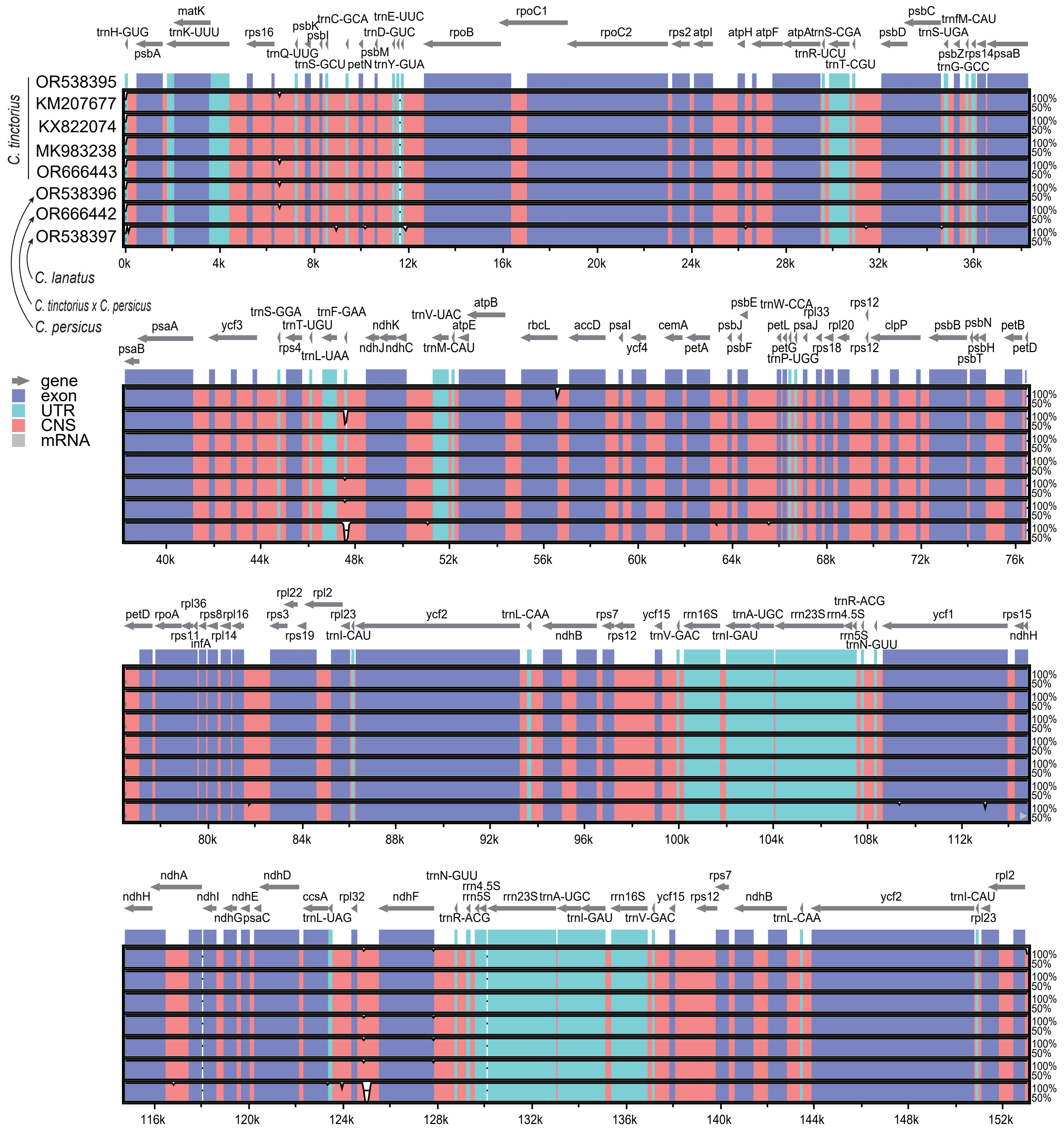

Figure S2. Comparative analysis of sequence differences in the chloroplast genomes of four *Carthamus* species with *C. tinctorius* (OR538395) as a reference.

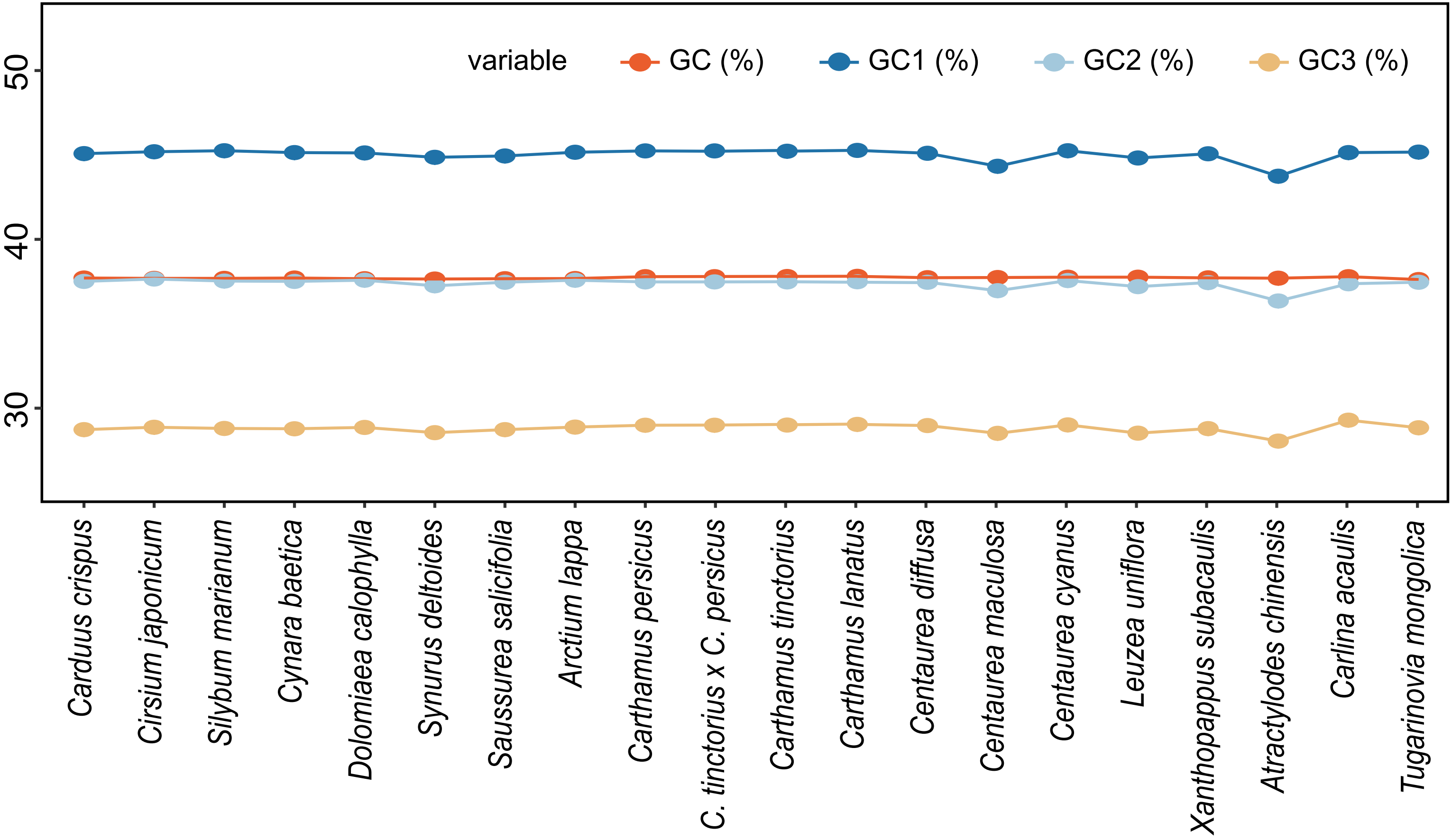

Figure S3. Statistical analysis of GC, GC1, GC2, and GC3 contents in the chloroplast genomes of 20 Cardueae species.
